# A115 IMPROVING ENDOSCOPY ROOM EFFICIENCY: EVALUATION OF A VIDEO AS A SUPPLEMENTARY TOOL FOR INFORMED CONSENT

**DOI:** 10.1093/jcag/gwae059.115

**Published:** 2025-02-10

**Authors:** A Kyei, O esenwa, C Tan, D Llovet, M Bernstein, B Mannino, L Cohen, N Griller, F Saibil, P Tartaro, E Yong, J Tinmouth

**Affiliations:** Sunnybrook Health Sciences Centre, Toronto, ON, Canada; University of Ottawa Faculty of Medicine, Ottawa, ON, Canada; University of Toronto Department of Medicine, Toronto, ON, Canada; Ontario Health, Toronto, ON, Canada; University of Toronto Department of Medicine, Toronto, ON, Canada; Sunnybrook Health Sciences Centre, Toronto, ON, Canada; Sunnybrook Health Sciences Centre, Toronto, ON, Canada; Sunnybrook Health Sciences Centre, Toronto, ON, Canada; Sunnybrook Health Sciences Centre, Toronto, ON, Canada; Sunnybrook Health Sciences Centre, Toronto, ON, Canada; Sunnybrook Health Sciences Centre, Toronto, ON, Canada; University of Toronto Department of Medicine, Toronto, ON, Canada

## Abstract

**Background:**

Endoscopy unit efficiency is critical because of the need to provide timely and quality care, despite limited resources. In previous work, obtaining informed consent negatively impacted efficiency. We developed a 3-minute animated video to facilitate the consent process, including describing colonoscopy, its purpose and potential risks/benefits.

**Aims:**

1) Assess the ability of the video to support the informed consent process; 2) Determine the effectiveness of the video as a communication tool.

**Methods:**

Using a critical case sample design with maximum variation, 12 participants completed pre- and post-colonoscopy 1:1 semi-structured interviews after viewing the video. Questions evaluated whether key components of informed consent were conveyed and assessed the video using principles of learner verification (attractiveness, usability, comprehension, impact on self-efficacy, acceptability). Interviews were recorded and transcribed. The data were coded inductively and deductively.

**Results:**

Regarding components of informed consent, most participants understood the purpose and nature of a colonoscopy, but alternatives, including the right to refuse, were less effectively communicated. As a communication tool, the animations engaged participants and aided comprehension of complex material. The language was accessible, however, some participants found the video too fast and the font too small. Most participants found the video acceptable and characters relatable. Some identified information gaps included sedation level and procedure duration.

**Conclusions:**

Endoscopy unit efficiency may be improved by providing consent information via video to patients scheduled for colonoscopy to supplement current approaches to informed consent. Our findings will inform revisions of the video and subsequent implementation into clinical practice.

**Funding Agencies:**

This study is being funded by the principal investigator’s own funds (Jill Tinmouth)

